# Role of Oxidative Stress in the Neurocognitive Dysfunction of Obstructive Sleep Apnea Syndrome

**DOI:** 10.1155/2016/9626831

**Published:** 2016-09-28

**Authors:** Li Zhou, Ping Chen, Yating Peng, Ruoyun Ouyang

**Affiliations:** ^1^Department of Respiratory Medicine, The Second Xiangya Hospital, Central South University, 139 Renming Middle Road, Changsha, Hunan 410011, China; ^2^Research Unit of Respiratory Disease, Central South University, 139 Renming Middle Road, Changsha, Hunan 410011, China; ^3^Treatment Center of Respiratory Disease, Central South University, 139 Renming Middle Road, Changsha, Hunan 410011, China

## Abstract

Obstructive sleep apnea syndrome (OSAS) is characterized by chronic nocturnal intermittent hypoxia and sleep fragmentations. Neurocognitive dysfunction, a significant and extraordinary complication of OSAS, influences patients' career, family, and social life and reduces quality of life to some extent. Previous researches revealed that repetitive hypoxia and reoxygenation caused mitochondria and endoplasmic reticulum dysfunction, overactivated NADPH oxidase, xanthine oxidase, and uncoupling nitric oxide synthase, induced an imbalance between prooxidants and antioxidants, and then got rise to a series of oxidative stress (OS) responses, such as protein oxidation, lipid peroxidation, and DNA oxidation along with inflammatory reaction. OS in brain could trigger neuron injury especially in the hippocampus and cerebral cortex regions. Those two regions are fairly susceptible to hypoxia and oxidative stress production which could consequently result in cognitive dysfunction. Apart from continuous positive airway pressure (CPAP), antioxidant may be a promising therapeutic method to improve partially reversible neurocognitive function. Understanding the role that OS played in the cognitive deficits is crucial for future research and therapeutic strategy development. In this paper, recent important literature concerning the relationship between oxidative stress and cognitive impairment in OSAS will be summarized and the results can provide a rewarding overview for future breakthrough in this field.

## 1. Introduction

Obstructive sleep apnea syndrome (OSAS), a clinical syndrome manifesting as repetitive episodes of partial or complete collapse of the upper airway during sleep, results in recurrent nocturnal apnea, chronic intermittent hypoxia (CIH), transitory hypercapnia, and sleep fragmentation. It has been recognized as a paramount and growing prevalent public health problem affecting 22% of men and 17% of women on average [1]. Among the population with OSAS, certain subgroups like middle-age adults and elderly stand at a higher risk [2], which is also closely associated with a series of adverse complications such as hyperlipemia [3, 4], type 2 diabetes [5, 6], cardiovascular disease [7–9] (systemic hypertension [10], coronary disease [11], heart failure [12], and stroke [13]), pulmonary hypertension [14], and neurocognitive deficits [15, 16]. Cognitive impairment in OSAS individuals is involved with various cognitive domains, such as attention/vigilance, memory, and global cognitive function as well as executive function. Substantial studies have proved that oxidative stress (OS), a consequence of chronic recurrent hypoxia and reoxygenation and an extraordinary feature of OSAS, plays a significant role in cardiovascular disease associated with OSAS. More importantly, accumulating evidence has demonstrated that OS is also one of the important mechanisms leading to neurocognitive dysfunction. This review will summarize the current research conducted neurocognitive impairment and OSAS and the role of oxidative stress in neurocognitive impairment of OSAS.

## 2. OS in OSAS

Oxidative stress (OS) is a state of prooxidant/antioxidant imbalance resulting from a variety of exogenous or endogenous stimulation or stress. It could be induced by overproduction of reactive oxygen species (ROS) and reactive nitrogen species (RNS) or a decreased capacity of antioxidant. There are two kinds of antioxidants. The first one is enzymatic processes, including superoxide dismutase (SOD), catalase (CAT), glutathione peroxidase (GSH-Px), peroxiredoxin, glutathione reductase, and thioredoxin reductase (TRXR). The other type of antioxidant includes ergothioneine, vitamin C, vitamin E, glutathione melatonin, alpha lipoic acid, carotenoid, copper, zinc, and selenium. Recent studies have suggested that the recurrence of the process of hypoxia/reoxygenation in OSAS contributes to the imbalance between antioxidant defense system and oxidant system, which could lead to OS and then activate and accelerate peroxidation damage and inflammation reaction. A series of transcription factors, such as hypoxia inducible transcription factors-1*α* (HIF-1*α*), nuclear factor-like 2 (Nrf2), activator protein 1 (AP1), and nuclear factor *κβ* (NF*κβ*), are activated in OSAS [17]. Consequently, damage to tissue and cells, endothelial dysfunction [18], and metabolic disturbance occurred, and other comorbidities such as type 2 diabetes, dyslipidemia, cardiovascular complications [19–23], and neurocognitive impairment followed [17, 24].

### 2.1. Biomarkers of OS Levels

OS is a result of imbalance of oxidative system and antioxidative system. The major OS damage includes lipid peroxidation, protein oxidation, RNA and DNA damage, protein nitration, production of ROS and peroxide, and change of total antioxidant capacity. Biomarkers related to OS include protein oxidation (protein carbonyl, advanced oxidative protein production, AGE, glutathione [25], GSSG, 3-nitrotyrosine), lipid peroxidation (8-hydroxyguanosine, thiobarbituric acid reactive substances, ox-LDL, malondialdehyde (MDA) [26], 4-hydroxynonenal, and LOOH), DNA damage (8-hydroxydeoxyguanosine [27], 8-hydroxyguanosine, and comet assay), antioxidation (SOD, CAT, and GSH-Px), ROS, nitrate, nitrite, NADPH oxidase (Nox), Cp, paraoxonase, arylesterase, sulfhydryl group, TAS, TOS, and OSI. In addition, previous research suggests that CCAT-enhancer binding protein (C/EBP) homologous binding protein (CHOP), which is indispensable for processes like NADPH oxidase subtype 2 (Nox2), ROS, and hypoxia inducible factor-1*α* activation (HIF-1*α*), might be an upstream target to protect OSAS patients from oxidative damage [28]. Furthermore, the concentration of ROS, RNS, and oxygen ion can be directly and accurately measured by an advanced method electron paramagnetic resonance with high sensitivity [29].

A multitude of studies have manifested the occurrence of oxidative stress in obstructive sleep apnea (OSA) patients [30–33]. A study by Lavie et al. [34] has investigated 114 OSA patients and 30 nonapneic controls. Among the OSA patients, 59 have cardiovascular disease (CVD). 55 have no cardiovascular disease (CVD). It was found that the level of paraoxonase-1 (PON1) is lower and the concentrations of thiobarbituric reactive substances (TBARS) and peroxides (PD) are higher in OSA patients group compared with control subjects. Besides, a negative association between PON1 activity and respiratory disturbance index (RDI) was observed, while TBARS and PD were significantly positively correlated with RDI. After nCPAP treatment, the levels of TBARS and PD were significantly decreased. Increased levels of other lipid peroxidation biomarkers, such as MDA and 8-isoprostane, were also observed in OSAS patients in other researches and improved after CPAP treatment [18, 35, 36].

Mancuso et al. [37] have assessed levels of advanced oxidation protein products (AOPP), ferric reducing antioxidant power (FRAP), and total glutathione (GSH) in two groups (41 OSA patients and 32 healthy control subjects). All the subjects are free of comorbidities and are nonsmokers. They found a significant increase in serum AOPP concentration and a decrease in FRAP and GSH levels in OSA patients. This study revealed that OSAS patients were subjected to protein oxidation and antioxidative capacity impairment. CPAP treatment improved the abnormal FRAP level, which suggests that FRAP is likely to be a potential biomarker to access OS level after CPAP treatment. A cross-sectional study measuring urinary excretion of 8-hydroxy-2′-deoxyguanosine (8-OHdG) as an indicator of DNA OS damage also showed similar results [27].

Hopps et al. [38] have measured concentration of TBARS and carbonyl between a group of 27 severe OSAS patients (AHI > 30) and a group of 21 mild-to-moderate OSAS patients (AHI < 30). Significantly higher levels of TBARS and carbonyl were found in severe OSAS group. Besides, these biomarkers were positively associated with neck and waist circumference, AHI value, and oxygen desaturation index, respectively, and negatively associated with the mean oxygen saturation. Similarly, Franco et al. [39] have reported significantly higher superoxide radical and lower levels of serum nitrates and nitrites in OSAS patients compared to healthy subjects in a severity-dependent manner. They also found that moderate and severe OSAS patients had semblable OS profile and condition, which were remarkably different from mild patients. That is, a critical condition probably exists in the triggering of oxidative stress metabolism and symptoms between mild and moderate OSAS.

### 2.2. Posttreatment OS Levels

A number of studies have suggested that treatment with CPAP could attenuate OS levels in OSA patients [35, 40–45]. Carpagnano et al. [46] have reported that serum and exhaled breath condensate concentration of 8-isoprostane were progressively decreased after CPAP treatment. Relevant conclusions in the population of elderly OSAS were also reached by Yagihara et al. [41], who reported significantly reduced MDA levels after six months of CPAP therapy. Similarly, Oyama et al. [43] found markedly raised plasma levels of nitric oxide and declined TBARS and asymmetrical dimethylarginine levels after three months of CPAP treatment. In recent years, the relationship between OS and pediatric OSA became a rising concern. Similar to adults, pediatric OSA is associated with an increased OS. The study conducted by Tauman found that OSA in children is associated with increased lipid peroxidation, which was positively correlated with disease severity and the degree of intermittent hypoxia [31].

### 2.3. Mechanism of OS in OSAS

There are several subcellular compartments involved in the production of ROS, such as mitochondria, endoplasmic reticulum, cellular membrane, lysosomes, peroxisomes, and the enzymatic systems which include NADPH oxidases (Nox2 and Nox4), xanthine oxidase (XOD), phospholipase A2, lipoxygenases, cyclooxygenase, and uncoupled nitric oxide synthases (NOSs) [27, 47, 48]. The mitochondria, which are main sources for the formation of ROS from electron transport chain (ETC), are susceptible to hypoxia. Recurrent ischemia/reoxygenation in OSAS patients could lead to dysfunction of mitochondria and endoplasmic reticulum and activation of Nox, which will cause overproduction of ROS and OS eventually [30]. XOD plays a crucial role in cellular oxidative status, detoxification of aldehydes, and oxidative injury in ischemia-reperfusion [49]. A case-control study including 43 OSAS patients and 43 age- and sex-matched subjects showed that plasma concentration of xanthine/hypoxanthine was significantly increased in OSAS patients and positively associated with age, AHI, and severity of the disease [50]. In addition, Ntalapascha et al. [25] evaluated OS levels extensively in a homogenous population of severe OSAS patients (AHI > 30). These patients have undergone no treatment, free of comorbidities or factors known to augment OS per se. Their study demonstrated that OSA may be related to elevated OS burden through protein oxidation-GSH/GSSG pathway.

However, it should be noted that the pathogenic role of OS in OSAS is still controversial. Some studies failed to demonstrate that OSAS is linked to increased oxidative stress [26, 51, 52]. An analysis by Simiakakis et al. recruited a group of 42 moderate to severe OSAS patients and 24 healthy control subjects and revealed that smoking, obesity, and gender play crucial roles in determining OS levels of OSAS patients [53]. Confounding factors, such as age, obesity, smoking, dietary habits, hypertension, diabetes, hyperlipemia, coronary heart disease, metabolic syndrome, and other concurrent comorbidities which might augment OS, could cause experimental errors and bias if the researchers did not exclude these factors. Despite the argument, the understanding that OS plays a crucial role in the development of OSAS has seemed to be an emerging consensus [33]. However, more large-scale multicenter randomized control trials with homogeneous population are needed to be conducted and confirm whether OS is involved in OSAS and its complications.

## 3. Neurocognitive Dysfunction in OSAS

Cognitive function is an important component of human advanced nervous function. It includes psychological process such as feeling, consciousness, reasoning, language, thought, intelligence, and learning. The risk factors of cognitive disorder consist of age, gender, smoking, alcohol drinking, obesity, hypertension, chronic heart disease, diabetes, metabolic syndrome, stroke, hypothyroidism, active psychiatric drug, apolipoprotein E epsilon 4 (APOE *ε*4) allele, Down syndrome, abnormal maxillofacial anatomy, family history, and OSA [54]. Neurocognitive impairment of OSAS, involved in patients of all age ranges [55], has an unfavorable impact on the patients' work productivity, quality of life, and social safety.

### 3.1. Classification of Neurocognitive Dysfunction in OSAS

A systematic meta-review has indicated that OSAS are associated with a broad range of neurocognitive deficits: attention/vigilance, executive function, delayed long-term visual and verbal memory, global cognitive function, and visuospatial/construction abilities [16, 54]. Generally, attention is the most common impaired cognition in OSAS subjects and it can be divided into three components: sustained, selective, and divided attention [56]. Several studies [57, 58] have demonstrated that drivers with OSA have decreased visual vigilance/sustained attention, which is closely associated with sleep fragmentation/disorder related daytime sleepiness and tiredness, and they have a greater risk of motor vehicle crashes compared to the healthy population. In addition, by using event-related-potentials (ERP), Gosselin et al. [59] showed that OSA patients have involuntary attention switching deficit. And attention/vigilance damage has been shown to be positively associated with the severity of OSA [60].

Memory roughly includes two categories: short-term and long-term memory. A number of studies have suggested that OSAS patients were involved in short-term and working memory deficit, which was probably correlated with hypoxia-related change in hippocampal impairment [61]. The specific subcomponent of memory impairment involved in OSA has been controversial. The research by Twigg et al. [62] has shown that verbal but not visual memory was impaired in OSA patients. Yet, it was found that OSA patients have mild visual cognitive dysfunction [63]. And a recent meta-analysis which included 42 researches revealed that verbal episodic memory and visuospatial episodic memory mainly representing the domain of immediate and delayed recall were disturbed in OSA in comparison to healthy group [64].

Executive function, including inhibition, shifting, updating, and generativity as well as fluid reasoning, is the most vulnerable part among the neurocognitive functions [65, 66]. Several meta-analyses demonstrated that all subdomains of executive function, especially working memory, phonological fluency, cognitive flexibility, and planning, have been impaired in OSA patients [67]. Meanwhile, CPAP treatment could improve some but not all executive functions in different degrees [67–70].

Nevertheless, due to heterogeneity in methodology, there are controversial opinions about the impaired cognitive domains in OSA. Rather than intelligence, attention, memory, and executive function are the most reported cognitive deficits. Furthermore, the current meta-analysis revealed that treatment with CPAP improved cognitive dysfunction, especially attention and executive function, in patient with OSA [67, 71]. However, it seems that impaired cognition could be partially reversed after CPAP treatment [71, 72].

### 3.2. OSA and Brain Tissue Abnormality

Using imaging technology, previous researches found that in OSA patients there are different degree changes in extensive brains tissues including cerebra grey, white matter, hippocampus, frontotemporal and occipital lobe, thalamus, and basal ganglion as well as part of cerebellum [73–78]. The preliminary study manifested that the most obviously changed area of brain morphology in OSA patients was hippocampus, a part of limbic system which edits learning and memory function, especially the storage of short-term memory [77]. In addition, the cerebral grey is closely associated with executive function. By diffusion tensor magnetic resonance imaging, previous research demonstrated that extensive white matter impairment happened in OSA patients, especially in axon-related brain tissue such as limbic system, pons, and frontotemporal and parietal cerebral cortex [79]. Joo et al. [80] evaluated the structural differences in gray matter between newly diagnosed male patients and healthy people using optimized voxel-based morphometry, an automated processing technique for MRI. Their data showed that the gray-matter concentrations of OSA patients were significantly reduced in extensive brain region, such as the gyrus rectus, frontal gyri, precentral gyrus, frontomarginal gyri, anterior cingulate gyri, insular gyrus, caudate nuclei, thalami, amygdalohippocampal temporal gyri, and the cerebellum. However, the total volume of brain is normal. Chan et al. [81] used high resolution 3-dimensional magnetic resonance images of the brain to analyze grey matter density and cerebral volume in children with and without OSA. Their results showed that significant negative correlations were found between the visual-fine motor coordination score and the ratio of grey matter volume to total brain volume. However, Algin et al. [82] found significantly lower NAA/Cr ratios in the frontal cortex and frontal white matter of OSAS patients using magnetic resonance spectroscopy (MRS) and no neurochemical changes on T2 relaxometry and diffusion weighted imaging (DWI). Besides, cognitive impairments were related with focal reductions of gray-matter volume in the hippocampus, posterior parietal cortex, and superior frontal gyrus. Meanwhile, cognitive function such as memory, attention, and executive function was significantly improved after treatment, in parallel with increased gray-matter volume in hippocampal and frontal structures [83].

### 3.3. The Mechanism Involved in Cognitive Dysfunction in OSA Patients

It has been believed that the main mechanisms regulating the development of cognitive complaints were hypoxemia and sleep fragmentation. A prospective cohort study by Shpirer et al. [84] demonstrated that attention defect was closely associated with intermittent hypoxemia, not sleep fragmentation. Executive function was not affected by the degree of hypoxia. In a study on population with matched degree of daytime sleepiness, age, gender, and educational level, Quan et al. [85] found that motor speed and processing speed performance were negatively correlated with oxygen desaturation, but attention and executive function were not related to hypoxemia degree. Besides, a literature review by Sateia presented that defects in general intellectual function and executive function were strongly linked to the degree of hypoxia [86]. Yet, disturbances in vigilance, alertness, and memory seem to possibly have correlation with sleep disruption. In a rat CIH model study, Kheirandish et al. [87] proved that nocturnal hypoxemia could lead to the impairment in the spatial working memory and the frontal cortex. Hippocampus regions of rats after exposures to chronic intermittent hypoxia were markedly injured.

On the other hand, nocturnal arousal, sleep disorder, and slow-wave and rapid-eye-movement sleep deprivation in OSAS patients lead to daytime somnolence. There is a close association between daytime sleepiness and cognitive dysfunction including decline in attention, memory, and visuospatial ability [88]. In a previous study, O'Brien et al. investigated children with primary snoring and discovered that snoring children showed poorer general cognitive function, language, and visual spatial ability compared with healthy children [89]. An international epidemiological survey in a population of 13057 subjects also reached a conclusion that sleep arousal disturbance was closely related to neuropsychological changes in OSA patients [90]. In addition, metabolic disturbance of lipid and protein could also cause cognitive impairment. And higher intelligence and younger age seem to have a protective effect on OSA-associated cognitive defects [55]. The concentration of serum insulin-like growth factor (IGF) was significantly decreased in the group of OSA children with cognitive complication compared to the group of OSA children with normal cognitive score [91]. Notably, studies demonstrated that carrier rate of APOE *ε*4 allele is obviously increased in OSA patients with neurocognitive impairment compared to normal cognition people, which indicated that cognitive decline in OSA was probably linked to heredity [92]. What is more, beyond the influence of covariates and apnea severity, the level of nocturnal cortisol was also possibly associated with neuropsychological function [93].

## 4. The Role of Oxidative Stress in the Development of Cognitive Dysfunction in OSA

**Figure 1:**
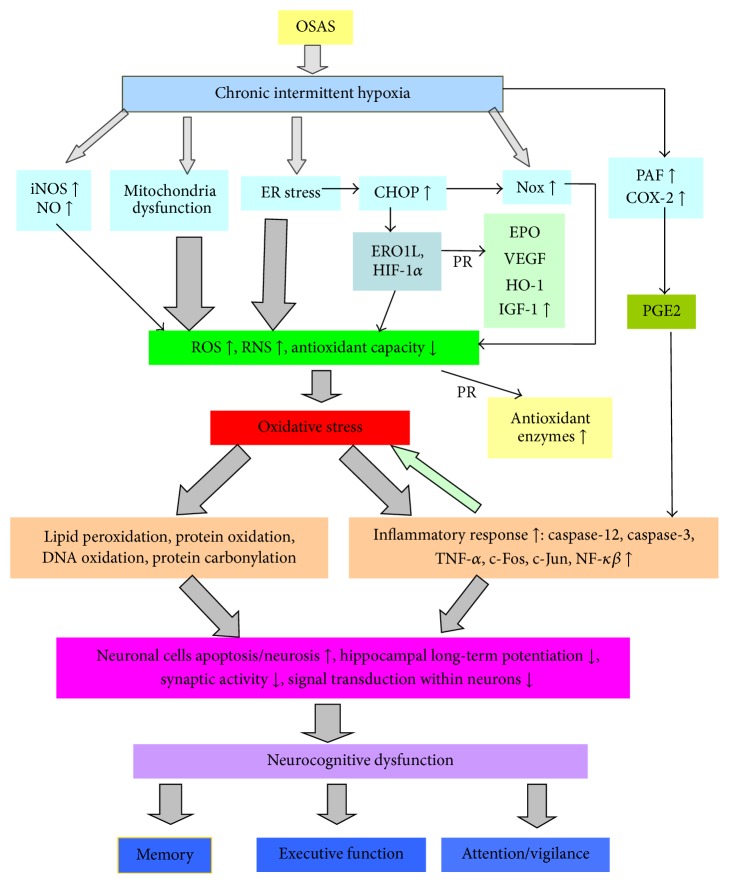
Schematic demonstration of the important role played by oxidative stress in the development of cognitive dysfunction in OSAS patients: chronic intermittent hypoxia (CIH) resulting from OSAS causes dysfunction of mitochondria and endoplasmic reticulum and overactivation of Nox, iNOS, PAF, and COX-2. All the above induce overproduction of ROS and RNS, as well as attenuated antioxidant capacity, and consequently contribute to imbalance of oxidation-antioxidation and a state of oxidative stress, which result in protein, lipid, and DNA peroxidation damage, and a series of inflammatory responses. Meanwhile, ER stress could upregulate CHOP expression, which could exacerbate production of ROS further. Substantial inflammatory cytokines and peroxidation lead to necrosis and apoptosis of nerve cell, which eventually results in gradual neurocognitive dysfunction of OSA patients. PAF: platelet-activating factor; Nox: NADPH oxidase; ERO1L: endoplasmic reticulum oxidoreductin-1-like; COX-2: cyclooxygenase-2; VEGF: vascular endothelial growth factor; HO-1: heme oxygenase-1; ER: endoplasmic reticulum; IGF: insulin-like growth factor; iNOS: inducible nitric oxide synthase; CHOP: C/EBP-homologous protein; PR: protective factor.

It is generally recognized that oxidative stress is closely associated with the formation and development of nervous system diseases such as Alzheimer disease, Parkinson's disease, and epilepsy, as well as endothelial dysfunction and cardiovascular disease (CVD) in OSA. Researches have indicated that OS also play a critical role in the intermittent hypoxia induced nervous injury [17, 30]. Repeated processes of airway obstruction and collapse during sleep of OSAS patients lead to nocturnal chronic intermittent hypoxia (IH), result in mitochondria and endoplasmic reticulum dysfunction, excessively activate NADPH oxidase, and decline antioxidant capacity, which further trigger overproduction of ROS and consequently initiate protein, lipid, and DNA peroxidation damage and inflammatory response since the cerebral cortex and hippocampus are vulnerable to OS. These changes could mediate apoptosis and necrosis of nerve cells and then contribute to neuropsychological alterations [17, 94]. The latest clinical meta-analysis showed that the mechanism involved in cognitive impairment in OSA patients could be as follows: CIH and other risk factors promoted inflammation, endothelial dysfunction, and oxidative stress of central nervous system, thus causing cerebral cortex, brainstem, or other brain region dysfunction, and lead to neurocognitive dysfunction eventually (Figure 1) [95].

### 4.1. Association within OS and Cognitive Dysfunction in OSAS Patients (Table 1)

There are several researchers who observed the correlation between OS and cognitive dysfunction in OSAS patients via measuring OS biomarkers levels and neurocognitive test scores. An observational, cross-sectional study in a group of 14 OSA patients and 13 controls was conducted by Sales et al. [96] to explore the relationship between cognitive dysfunction and oxidative stress. They performed the Toulouse-Piéron Attention Test, Wisconsin Card Sorting Test (WCST), the Digit Symbol Substitution Test, the Forward Digit Span, the Similarities Test, the Logical Memory, Verbal Paired Association Tests, and the Rey-Osterrieth Complex Figure Test to evaluate various subcomponents of cognitive function. The concentrations of potential biomarkers for OS such as serum SOD, catalase, GSH, and vitamins were also evaluated. Their data revealed a positive correlation between vitamin E levels and performance in the Backward Digit Span task. And after matching age and body mass index, the correlation also remained. The concentrations of SOD correlated with the levels of executive nonperseveration errors in the Wisconsin Card Sorting Test, which suggested that an imbalance between antioxidants and prooxidants might induce the cognitive dysfunction of OSA patients. Yu et al. [9, 97] compared the differences in levels of serum MDA, SOD, and NO between OSAHS and healthy subject and analyzed the correlation between those markers and hypoxia index, apnea hypopnea index, and cognitive function scores separately. Their results showed that it is possible that hypoxemia and sleep fragmentation could cause overproduction of MDA and NO as well as decrease of antioxidation power to some degree, consequently making the patient be in a state of OS, and induce damage to nervous system, which is the biochemical foundation of cognitive dysfunction in OSA. Moreover, Huang et al. [98] used the mini-mental state examination (MMSE) and Montreal Cognitive Assessment (MoCA) to assess cognitive status and, concurrently, measured Nox activity and 8-OHdG level in OSA patients and healthy people. The correlation analysis showed that OS was likely to be one of the pathogeneses of cognitive complains in OSA. In addition, Yang et al. [99] measured the concentration of AOPP, SOD, and MDA in serum and analyzed their correlation with MMSE, clock drawing test, AHI, and the lowest SaO2. Their study revealed that the levels of biomarkers of OS were associated with the MMSE score and clock drawing test. All those clinical trials provide us with an original prospect to perform further studies and more randomized control trials to confirm our viewpoint. Nevertheless, on the other hand, as we can see from all the clinical studies, it is usually difficult for researchers to obtain patients' brain tissue to observe or detect its damage directly and distinctly. Thus, these clinical trials could not establish a direct effect relationship between neuropsychological dysfunction and OS. Because of limitation in obtaining the human body specimen, how to detect the level of cerebral OS and analyze association with cognitive disturbance in OSAS patients is still a dilemma. Some radiological technologies such as molecular imaging technology, functional magnetic resonance imaging (MRI), single photon emission computed tomography (SPECT), and optical imaging methods are promising to evaluate OS extent.

### 4.2. OS Leading to Cognitive Deficits in OSA Animal Model (Table 2)

So far animal models have been used to explore the relation between OS and cognitive deficits in OSA. Significantly elevated OS levels were detected in the hippocampus and cortex regions of chronic intermittent hypoxia (CIH) mice. Wang et al. [100] observed that there was obvious difference in apoptosis of neurocyte and HIF-1*α* expression in rats under hypoxia and normoxia condition. Three different groups of rats were included in the study: normoxia, intermittent hypoxia (IH), and continuous hypoxia (CH). The group of IH rats showed the highest percentage of apoptotic neuronal cells and HIF-1*α* expression. Besides, apoptotic neurons and HIF-1*α* expression mainly were distributed in the cerebral cortex and hippocampus. Similarly, the study by Xu et al. [101] showed that both ROS production and OS biomarkers in cortex and cortical neuronal cells of mouse brain were significantly increased upon exposure to CIH, followed by increased levels of protein oxidation, lipid peroxidation, and nucleic acid oxidation in mice brain cortex. Moreover, a lower level of steady-state ROS production and reduced number of neuronal apoptoses were detected in brain cortex of transgenic mice overexpressing Cu and Zn superoxide dismutase when exposed to CIH conditions compared to control mice. The increased ROS production and oxidative stress induced CIH-mediated cortical neuronal apoptosis and neurocognitive dysfunction. In addition, Row et al. [102] conducted a randomized controlled trial by two variable factors: oxygen concentration and injection of antioxidant PNU-101033E (PNU). Their experiment showed that CIH rat without PNU-101033E treatment had the worst cognitive function and the highest levels of lipid peroxidation and oxidant stress in brain tissue, and the antioxidant, PNU-101033E, attenuates the spatial learning dysfunction in the rats exposure to IH. These findings demonstrated that oxidative stress might play an important role in the neuron cell damage and consequent behavioral impairments associated with CIH.

Compared with other parts of body, brain needs higher energy consumption and oxygen so it is more sensitive to hypoxia. After exposure to CIH, malfunction of self-adjusting mechanism to hypoxia in human body starts to develop, followed by mitochondria dysfunction which leads to production of ROS. Shan et al. [103] analyzed the cellular mechanism of enhanced production of ROS during cortical neuronal cell damage and neurocognitive impairment using* in vitro* cultured cells and CIH mice models. Their data revealed that the neuronal cell loss and development of neurocognitive defects in OSA are mediated, in part, by CIH-mediated mitochondrial oxidative stress. In addition, they found that overexpression of manganese superoxide dismutase (MnSOD) in mitochondrion could reduce CIH-mediated cortical neuronal apoptosis and attenuate spatial learning deficits.

On the other hand, excessively activated Nox is also likely to play a vital role in the evolution of central nervous system dysfunction. Nox, specifically located in the membranes of phagocyte, is one of the key enzymes to produce ROS. When exceedingly activated, Nox induces oxidative stress. Nair et al. [104] observed spatial learning capacity difference between mice lacking Nox activity (gp91phox−/Y) and wild-type littermates exposed to IH. Significantly increased expression levels and activity of Nox as well as MDA and 8-OHDG were observed in cortical and hippocampal lysates of wild-type mice following IH exposures while remarkable spatial learning deficits were observed in those mice. Similarly, Zhan et al. [105] demonstrated that the gene and protein expression levels of Nox mediated by long-term hypoxia/reoxygenation in wake-active brain regions were obviously higher in wild-type mice compared to the transgenic Nox-knockout mice and mice with pharmacologic inhibition of Nox activity. These findings provide evidence to the concept that oxidative stress responses induced by overactive Nox play a crucial role in the neurobehavioral impairments induced by IH during sleep.

Meanwhile, thioredoxin (Trx), as an antioxidase, could reduce levels of ROS and concentration of protein thiols. Yang et al. [106] examined mRNA and protein expression of Trx in the hippocampus tissue and the number of apoptotic cells in the hippocampus CA1 region. They found declined Trx mRNA and protein levels in the CIH-hippocampus of rats exposed to CIH and an elevated apoptosis percentage in hippocampal neurons. And apoptotic index (determined by counting the percentage of TUNEL-positive cells/high-power field (×100) in at least five high-power fields) of the neurons in the hippocampus was negatively associated with mRNA levels and protein expression of Trx. They suggested that lower level of Trx may play an important role in the impaired cognition in rats exposed to CIH through inducing apoptosis of neurons in the hippocampus. It has also been confirmed that cyclooxygenase-2 is upregulated in the neurological disorder, such as ischemic brain injury, Alzheimer disease, and stroke. IH-induced OS and proinflammatory cytokines may mediate upregulation of the RNA and protein expression levels of COX-2 and substantial increase of prostaglandin E2 (PGE2), thereby leading to spatial learning deficits in OSA. And COX-2 inhibitor NS-398 attenuated neuron apoptosis and neurobehavioral disturbance in rodent CIH model [114].

CHOP, a transcription factor, and a major mediator of ER stress-induced apoptosis signaling pathways, regulates ROS formation [117]. Moderate CHOP may protect neuron from OS in OSA. Chou et al. [28] observed the association between CHOP and LTIH oxidative injury in the hippocampus and cortex via contrasting neuron oxidation and apoptosis in CHOP null and wild-type mice. Their data revealed that endogenous CHOP positively upregulated Nox2 and HIF-1*α* expression and this resulted in injury of brainstem motoneurons, cortex, and hippocampus, which might contribute to neurobehavioral impairments. What is more, apolipoprotein E (ApoE) could also attenuate OS induced neuron injury [118]. ApoE-deficient mice exhibited increased vulnerability to intermittent hypoxia induced spatial learning deficits [107, 118].

Furthermore, certain substance or factors could protect brain regions from OSA-associated neuronal impairment. Mice deficient of cell surface receptor platelet-activating factor (PAF), a bioactive mediator of OS and inflammation, showed declined cyclooxygenase-2 and inducible nitric oxide synthase activities and spatial learning deficits associated with IH [108]. The study by Dayyat et al. [109] demonstrated that exogenous administration of erythropoietin (EPO) attenuated OS and neurocognitive damage in murine model of OSA. Their research indicated that it might be promising to stop the involution or potentially reverse cognitive morbidities in OSA by either increasing EPO expression or the activation of EPO receptors in the CNS. Recently Nair et al. [110] found that in mice model treatment with growth hormone releasing hormone (GHRH) agonist JI-34 can weaken IH-induced neurocognitive deficits, decrease oxidative stress levels, and increase HIF-1*α* DNA binding and upregulation of IGF-1 and erythropoietin expression, while GHRH antagonist (MIA-602) did not affect any cognitive disorders in OSA mice. Furthermore, Li et al. [111] proved that administration of exogenous growth hormone (GH) not only upregulated the hippocampal mRNA expression of IGF-1, EPO, and VEGF but also consequently reduced IH-induced hippocampal injury as well as cognitive deficits. Studies confirm that telmisartan, an angiotensin II type 1 receptor blocker (ARB), can be beneficial for adjusting the levels of nitric oxide and nitric oxide enzyme, which play important roles in attenuating oxidative stress, anti-inflammatory response, and suppressing neural apoptosis. Thus, Yuan et al. [112] proved that iNOS was overexpressed in the hippocampus of CIH mice and telmisartan reduced the iNOS level; therefore telmisartan has a protective effect on hippocampal apoptosis induced by CIH. In addition, a study found that high fat diet may increase OS damage, cause damage in hippocampal CA1 area, and then lead to cognitive dysfunction [113]. Notably particularly, Burckhardt et al. [115] found that green tea catechin polyphenols (GTPs), a common biologically active compound present in green tea, not only attenuated IH-induced oxidative stress and inflammatory load in the cortex and hippocampal CA1 region of model rat brain but also improved IH-induced spatial learning deficits. Resveratrol, a natural polyphenolic compound which exists in the skin and seeds of plants, such as grapes, grains, berries, peanuts, and red wine, has been proved to increase the expression of antioxidant enzymes and has a neuroprotective effect to many neurodegenerative diseases [116]. The latest research revealed that resveratrol could also prevent IH-induced spatial memory deficits via reducing activity of the hippocampal oxidative stress pathways and the expression of p47Phox subunit of NADPH oxidase [116]. Those two studies provided hopeful therapeutic measures in improving cognitive dysfunction of OSA patients.

## 5. Conclusion

Repetitive episodes of obstruction of the upper airway induce chronic intermittent hypoxia, then cause dysfunction of mitochondria, endoplasmic reticulum, and endothelium, compromised energy metabolism, and activation of Nox, xanthine oxidase, and iNOS, consequently contributing to overproduction of ROS and imbalance of oxidation-antioxidation, lead to a state of OS, which produces protein, lipid, and DNA peroxidation damage, and result in substantial inflammatory response. However, cerebral neural cells, especially in the regions of hippocampus and cerebral cortex, are susceptible to hypoxemia. CIH-induced OS could lead to necrosis and apoptosis of nerve cell, which results in gradual neurocognitive dysfunction of OSA patients, presenting short-term declined attention and vigilance and long-term degeneration of memory as well as executive function. In addition to CPAP treatment, experiments in CIH animal models demonstrated that administration of antioxidant such as EPO, GH, JI-34, NS-398, or telmisartan might provide a method to protect IH-vulnerable brain regions from OSA-associated neuronal damage and neurocognitive dysfunction. However, either CPAP treatment or antioxidant administration methods have shown direct evidence verifying relationship between oxidative stress and neurocognitive dysfunction in OSA patients. And the effect of these two methods has not yet been confirmed by clinical trials. Moreover, specific upstream or downstream signaling pathways and the molecular mechanism underlying OS induced cognitive impairment are still not clear and need to be investigated further.

## Figures and Tables

**Table 1:** Association between OS and cognitive dysfunction in OSAS patients.

Reference	E group	C group	OS biomarkers lever	Cognitive test	Cognitive function	Relevance
Sales et al., 2013 [96]	14 male OSA	13 male subjects	Lower level of VitE, SOD, and VitB11 and higher homocysteine. Unchanged VitC, catalase, glutathione, and VitB12 level	WCST, the Digit Symbol Substitution Test, Digit Span, the Similarities Test, the Logical Memory and Verbal Paired Association Tests, and the Rey-Osterrieth Complex Figure Test	Worse attention, working memory, and verbal memory performance	Showing correlation between SOD, VitE, and cognitive function

Li et al., 2014 [9]	28 OSAS	16 healthy adults	Significantly reduced serum SOD concentration and increased MDA concentration in OSAHS	MoCA	Delay recall, calculation, and language were impaired in OSAS	Serum SOD and MDA level were correlated with impaired neurocognitive function

Li and Qin, 2007 [97]	18 OSAS	14 healthy adults	Increased serum NO concentration	WMS-RC, WAIS-RC	Impaired memory (visual recognition and digit symbols)	The NO concentration was negatively related to cognitive function

Huang et al., 2014 [98]	41 OSAHS	44 healthy adults	Higher Nox activity and serum 8-OHdG concentration in OSAHS	MMSE, MoCA	Impaired delay recall, attention, language, visual spatial, and executive function in OSAHS	Cognitive function was negatively associated with the Nox activity and serum 8-OHdG level

Yang et al., 2013 [99]	67 OSAHS	20 healthy adults	Elevated AOPP, MDA and reduced SOD level in OSA patients	MMSE, ESS, and CDT	Impaired attention, calculation, and memory	AOPP, MDA, and SOD concentration were associated with the MMSE and CDT score

ESS: Epworth sleepiness scale; MMSE: mini-mental state examination; CDT: clocking drawing test; MoCA: Montreal Cognitive Assessment; WMS-RC: Wechsler memory scale-revised in China; WAIS-RC: Wechsler adult intelligence-revised in China; MDA: malondialdehyde; SOD: superoxide dismutase; AOPP: advanced oxidation protein products.

**Table 2:** The role of OS in the neurocognitive deficits of OSA animal model.

Reference	E group	C group	Detecting parameter	Morris water maze testing	Outcome
Wang et al., 2010 [100]	Male Wistar mice + IH	Male Wistar mice + RA; male Wistar mice + CH	Apoptotic neuronal cell, HIF-1*α* protein, and RNA	NA	HIF-1*α*↑ distributing with neuron apoptosis consistently in brain cortex and hippocampus of E group

Xu et al., 2004 [101]	Transgenic mice overexpressing SOD + IH; C578L/6J mice + IH	Transgenic mice overexpressing SOD + RA; C578L/6J mice + RA	ROS production, c-Fos, c-Jun, NF-*κβ*, caspase-3, carbonyl protein, MDA, 8-hydroxyguanosine, and neuronal cell apoptosis	Spatial task acquisition↓, working spatial memory↓	All the parameters increased in brain cortex upon CIH-C578L/6J mice; transgenic mice showing lower level compared with NCM

Row et al., 2003 [102]	V-IH; PNU-IH	V-RA; PNU-RA	MDA, isoprostane, and oxo8dG/oxo8G	The longest latencies and path lengths to locate the hidden platform in V-IH	The highest MDA, isoprostane, and oxo8DG/oxo8G in the cortex and hippocampal CA1 region of V-IH. PNU-101033E decreased OS level and improved neurocognitive deficits

Shan et al., 2007 [103]	(1) Transgenic mice overexpressing SOD + IH; C578L/6J mice + IH (2) Cortical neurons + CIH	(1) Mice + RA (2) Cortical neurons + RA	ROS production in cortical neurons, MDA, and protein oxidation	Reduced spatial learning deficits in the mice exposure to CIH	Elevated ROS production in cortical neuronal cortex and apoptotic neuronal cell. Transgenic mice showing reduced cortical neuron apoptosis and ROS production

Nair et al., 2011 [104]	gp91phox−/Y mice + IH; C578L/6J mice + IH	gp91phox−/Y mice +RA; C578L/6J mice + RA	NADPH oxidase expression and activity, MDA, and 8-OHDG	Spatial learning and memory deficits showing in IH-C57BL6/J mice, not in gp91phox−/Y mice exposed to IH	All the parameters were significantly increased in IH-C57BL6/J mice in the cortex and hippocampus. Nox activities were attenuated in gp91phox−/Y mice

Zhan et al., 2005 [105]	gp91phox−/− mice + IH; C578L/6J mice + IH	Mice + sham LTIH (normal Sp02)	NADPH oxidase gene and protein responses; p67phox, TNF-*α*, iNOS, COX-2 gene; protein carbonyl; F2 isoprostanes	NA	All the parameters showing increase in wide-type mice exposed to LTIH in wake-active region of the brain; transgenic absence and inhibiting NADPH oxidase activity showing declined OS damage

Yang et al., 2012 [106]	CIH + NS group; CIH + NAC group	Sham CIH + NS group; sham CIH + NAC group	Expression of Trx mRNA and protein, cells apoptosis in the hippocampus CA1 region	Impaired spatial learning and memory in CIH-rats	CIH rats showing decreased Trx mRNA and protein levels and elevated apoptotic cells in the hippocampus

Chou et al., 2013 [28]	CHOP null adult male mice + LTIH; wild-type adult male mice + LTIH	CHOP null + sham LTIH; wild-type adult male mice + sham LTIH	Nox2, CC-3, MAP-2, ChAT, and ERO1L in motor nuclei, CHOP; protein oxidation; neuronal apoptosis	NA	Relative to wild-type mice, CHOP−/− mice prevent oxidative stress (superoxide production/carbonyl proteins), neuronal apoptosis, and upregulation of Nox and HIF-1*α* in brain regions of cortex, hippocampus, and brainstem motoneurons

Kheirandish et al., 2005 [87, 107]	ApoE−/− mice, wild-type littermates in IH	ApoE−/− mice, wild-type littermates in RA	Prostaglandin E2 and MDA in hippocampal region	Longer times (latency) and distances (pathlength) to locate the hidden platform in IH mice	The highest PGE2 and MDA concentrations presenting in hippocampal brain tissues of ApoE−/− mice exposed to IH

Row et al., 2004 [108]	PAFR–/– mice, wild-type littermates in IH	PAFR–/– mice, wild-type littermates in RA	NOS activity, PGE2, COX-2, proteasomal activity, and CC-3	PAFR–/–mice in CIH displaying normal spatial learning compared with wild-type littermates	All the parameters showing increase in prefrontal cortex and the hippocampus CA1 region of wide-type mice exposed to IH. PAFR−/− mice showing attenuated OS

Dayyat et al., 2012 [109]	(1) V-IH; EPO-IH (2) Primary neuronal cell cultures	(1) V-SH; EPO-SH (2) V-RA; EPO-RA	NADPH oxidase, MDA, 8-OHDG, and EPO	EPO-IH mice showing normal learning. V-IH mice displaying spatial learning deficits	V-IH mice, but not EPO-treated IH-exposed mice, showing elevated levels of NADPH oxidase expression, MDA, and 8-OHDG in cortical and hippocampal lysates

Nair et al., 2013 [110]	V-IH; JI-34-IH	V-RA; JI-34-RA	MDA, 8-OHDG, HIF-1*α* DNA, EPO, and IGF-1 expression	JI-34 attenuated spatial learning performance deficits in mice exposed to IH	V-IH mice showing increased MDA and 8-OHDG in hippocampus and cortex; JI-34 reduced OS and increased HIF-1*α* DNA binding and expression of IGF-1 and EPO

Li et al., 2011 [111]	V-IH; GH-IH	(1) V-RA; GH-RA (2) CH	EPO, VEGF, HO-1, and GLUT-1 mRNA expression	GH attenuated IH-induced neurocognitive deficits	GH increased mRNA expression of IGF-1, EPO, and VEGF in the hippocampus

Yuan et al., 2015 [112]	V-IH; telmisartan-IH	V-RA; telmisartan-RA	MDA, NOS activity, NO content, and apoptotic cells in hippocampus; plasma CRP and IL-6	NA	Increased iNOS, NO content, MDA, and inflammatory reaction showing in the hippocampus of IH mice. Telmisartan attenuated above response and apoptosis in hippocampus

Goldbart et al., 2006 [113]	HF/RC + IH; LF/CC + IH	HF/RC + RA; LF/CC + RA	CREB phosphorylation in the CA1 region of the hippocampus	The worst place-training reference memory task deficits occurring in HF/RC + IH mice	Abundant reduced CREB phosphorylation showing in CA1 of IH mice

Li et al., 2003 [114]	V-IH; NS398-IH	(1) V-RA; NS398-RA (2) V-CH	COX-1 gene, COX-2 genes and protein expression and activity, and PGE2 concentration in cortical regions of rat brain	Deficits in the acquisition and retention of a spatial task showing in IH mice. NS-398 treatment attenuated IH-induced neurobehavioral deficits	Increased COX-2 protein and gene expression, PGE2 levels, and neuronal apoptosis in cortex

Burckhardt et al., 2008 [115]	V-IH; GTP-IH;	V-RA; GTP-RA	MDA, PGE2, p47phox mRNA, GFAP, RAGE, and the ratio of RAGE/*β*-actin in the cortical and hippocampal regions of rat model	GTPs are capable of attenuating IH-induced spatial learning deficits	All parameters showed increases in the brain cortex and hippocampus of IH-exposed rats. GTPs attenuated IH-induced oxidative stress and inflammatory reaction damage in the rat brain

B. A. Abdel-Wahab and M. M. Abdel-Wahab, 2016 [116]	V-IH; resveratrol-IH	V-RA; resveratrol-RA	TBARS, GSH, glutamate, GSH-Px activity, 8-OHdG, total protein, and p47phox mRNA in the hippocampus	Resveratrol protects animals from IH-induced spatial memory deficits	Resveratrol prevented IH-induced increases of glutamate, TBARS, and 8-OHdG levels and p47Phox expression in the hippocampus of IH rats and decreases of hippocampal GSH levels and GSH-Px activity

8-OHDG: 8-hydroxydeoxyguanosine; MDA: malondialdehyde; PGE2: prostaglandin E2; NOS: nitric oxide synthase; MAP-2: microtubule associate protein-2; ChAT: choline acetyltransferase; CC-3: cleaved caspase-3; Nox: NADPH oxidase; ERO1L: endoplasmic reticulum oxidoreductin-1-like; COX-2: cyclooxygenase-2; VEGF: vascular endothelial growth factor; HO-1: heme oxygenase-1; CREB: cyclic AMP response element binding protein; PNU: PNU-101033E; oxo8DG/oxo8G: 8-hydroxy-2′-deoxyguanosine/8-hydroxyguanosine; COX: cyclooxygenase; Trx: thioredoxin; ApoE: apolipoprotein E; GFAP: glial fibrillary acidic protein; RAGE: receptor for advanced glycation end products; TBARS: thiobarbituric acid reactive substances; GSH: glutathione; GSH-Px: glutathione peroxidase; GTPs: green tea catechin polyphenols.

E group: experiment group; C group: control group; CIH + NS group: CIH + normal saline; (CIH + NAC) group: N-acetylcysteine-treated CIH; sham CIH + NS: a sham CIH group; CIH + NAC group: sham NAC-treated sham CIH; EPO-IH: exogenously erythropoietin treated IH; HF/RC + IH: high fat/refined carbohydrate diet + IH; LF/CC + IH: low fat/complex carbohydrate diet + IH.

V-IH: vehicle + IH; ApoE−/−: ApoE-deficient mice; PAFR–/–: PAFR-deficient mice.

IH: intermittent hypoxia; RA: room air; CH: continued hypoxia; LTIH: long-term intermittent hypoxia; sham LTIH.

NA: not administrated.
